# Enhanced imaging workflow for anatomic pathology: implementation of a knowledge actor

**DOI:** 10.1186/1746-1596-8-S1-S41

**Published:** 2013-09-30

**Authors:** Jacques Klossa, Thomas Schrader, Christel Daniel

**Affiliations:** 1 TRIBVN, France; 2 University of Applied Sciences Brandenburg, Germany; 3 Assistance-Publique Hôpitaux de Paris, France

## Introduction

In Pathology, the development of slide scanners offers the possibility to acquire whole slide images (WSI) from specimen stored on glass slides and this is the beginning of a new paradigm in this medical domain. Two DICOM supplements were defined by the DICOM WG26 in order to store and display such big amount of data. These WSI images are mainly used for teaching, for research and for collaborative and cooperative applications. However, automated integration in the hospital healthcare enterprise (IHE) needs much more detailed information for i) integrating the digitalization process into the general diagnostic process in a Pathology Department, in other words, to define the WSI "acquisition modality work list", and ii) integrating efficiently image analysis into the diagnostic process, e.g. to create "evidences" from a WSI.

## Methods

A pathologist who reads a slide uses an iterative process which includes exploration and analysis phases, each at varied magnification. In comparison, a slide scanner acquires the whole slide or a pre-defined region of interest. In practice, we know that medical questioning may require more sophisticated possibilities like combining low RGB resolution scan (e.g. 5x to 20x) with higher resolutions and multi-z imaging in some specific region which could be addressed by some slide scanners provided one can build the corresponding modality (ies) work list. The same issues happen for "evidence creation" through an image analysis workflow. Workflow can become even more complex when using a multimodal scanner which adds to the current modalities (RGB bright field and fluorescence) complementary ones like quantitative phase imaging for label free unstained samples and Raman micro-spectroscopy for molecular signature acquisition.

We have 2 choices for solving such issues: either the process is embedded in the scanner software inside a proprietary solution, or the process can be driven by a scenario following the medical needs and assigns to each modality unitary task to be executed.

## Results

Let us try to scenarize the fairly simple use case of "Malaria". Following the current guidelines, diagnostics and personalized care aims at parasitemia counting and species identification on red blood cells (RBC): step 1 of the workflow asks for the acquisition of 200 fields at 100x and step 2 asks for individual classification of the retrieved infected RB cells. Such description is medically oriented and needs to be traduced in unitary technical tasks. Clearly, there is a need for some additional knowledge that explains how to select the fields to acquire, how to identify infected RBC and what information should be acquired on each RBC: color, MS images with or without z stack.

## Discussion

We are in favor of implementing in the workflow the second solution that avoids proprietary solutions and which could be achieved by adding to the IHE workflow a knowledge actor (KA) that will produce and orchestrate the scenario. KA would ask to the order filler patient and case information's in addition to the diagnostic questions. Based on the corresponding guidelines, the KA could then orchestrate a sequence of unitary work lists which can be executed by the microscopy scanner in conjunction with the evidence creator actor. Such solution would clearly separate medical guidelines from modality embedded software making the workflow more scalable.

Trying to benefit from previous IHE integration, we would suggest implementing such an actor in a way similar to the Treatment Management System (TMS), an information system that manages oncology information and is responsible for the scheduling of radiotherapy activities.

## Competing interests

The authors declare that they have no competing interests

## Authors’ contributions

• CD, TS and JK have developed the need for a new actor in the IHE workflow to answer the issue associated with modality driving and evidence creation

